# Polymyxin B‐Triggered Assembly of Peptide Hydrogels for Localized and Sustained Release of Combined Antimicrobial Therapy

**DOI:** 10.1002/adhm.202101465

**Published:** 2021-09-14

**Authors:** Yejiao Shi, David W. Wareham, Yichen Yuan, Xinru Deng, Alvaro Mata, Helena S. Azevedo

**Affiliations:** ^1^ School of Engineering and Materials Science Queen Mary University of London London E1 4NS UK; ^2^ Institute of Bioengineering Queen Mary University of London London E1 4NS UK; ^3^ Center for Immunobiology The Blizard Institute Barts and The London School of Medicine and Dentistry Queen Mary University of London London E1 2AT UK; ^4^ Barts Health NHS Trust London E1 2AT UK; ^5^ School of Pharmacy University of Nottingham Nottingham NG7 2RD UK; ^6^ Department of Chemical and Environmental Engineering University of Nottingham Nottingham NG7 2AT UK; ^7^ Biodiscovery Institute University of Nottingham Nottingham NG7 2RD UK

**Keywords:** antimicrobial, combined therapy, hydrogels, local delivery, peptide amphiphiles, polymyxins

## Abstract

Repurposing old antibiotics into more effective and safer formulations is an emergent approach to tackle the growing threat of antimicrobial resistance. Herein, a peptide hydrogel is reported for the localized and sustained release of polymyxin B (PMB), a decade‐old antibiotic with increasing clinical utility for treating multidrug‐resistant Gram‐negative bacterial infections. The hydrogel is assembled by additing PMB solution into a rationally designed peptide amphiphile (PA) solution and its mechanical properties can be adjusted through the addition of counterions, envisioning its application in diverse infection scenarios. Sustained release of PMB from the hydrogel over a 5‐day period and prolonged antimicrobial activities against Gram‐negative bacteria are observed. The localized release of active PMB from the hydrogel is shown to be effective in vivo for treating *Pseudomonas aeruginosa* infection in the *Galleria mellonella* burn wound infection model, dramatically reducing the mortality from 93% to 13%. Complementary antimicrobial activity against Gram‐positive *Staphylococcus aureus* and enhanced antimicrobial effect against the Gram‐negative *Acinetobacter baumannii* are observed when an additional antibiotic fusidic acid is incorporated into the hydrogen network. These results demonstrate the potential of the PMB‐triggered PA hydrogel as a versatile platform for the localized and sustained delivery of combined antimicrobial therapies.

## Graphical abstract

The decade‐old antibiotic polymyxin B is revitalized into a hydrogel formulation for the local and effective treatment of Gram‐negative bacteria infections. A simple and versatile peptide assembly approach is utilized to formulate the hydrogel, which allows sustained antibiotic release and prolonged antibacterial efficiency, while offering the possibility for combined antimicrobial therapy.

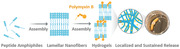

## 1 Introduction

Antibiotics remain the main agent to treat bacterial infections, even though their efficacy is now compromised due to overuse in humans, animals, and agriculture, with bacteria developing resistance that renders them ineffective.^[^ ^1^ ^]^ The increasing number of antibiotic‐resistant bacteria has emerged as a significant global concern and tackling the problem will require the discovery of novel antimicrobial agents and advanced therapies.^[^ ^2^ ^]^ However, with very few antibiotics in the pipeline and given the difficulties in bringing new antibiotics to the market,^[^ ^3^ ^]^ revitalizing the existing antibiotics and repurposing them into more effective and safer formulations may provide an alternative life‐saving therapy for immediate clinical applications.^[^ ^4^ ^]^

Polymyxins are a family of antibiotics, consisting of polymyxin B (PMB) and polymyxin E (also known as colistin), discovered more than 50 years ago.^[^ ^5^ ^]^ They are cyclic pentacationic lipopeptides that exert their antimicrobial activities by specifically binding to the anionic lipopolysaccharide within the outer membrane of Gram‐negative bacteria first, followed by penetrating into the outer membrane and leading to the bacterial cell death.^[^ ^6^ ^]^ Even though the clinical use of polymyxins was largely abandoned in the 1970s, because of their potential nephrotoxicity and neurotoxicity, they are increasingly used now by clinicians as the last resort to treat infections caused by multidrug‐resistant (MDR) Gram‐negative bacteria.^[^ ^7^ ^]^ However, due to the low oral bioavailability of PMB, only topical (ointment) and intravenous formulations are currently available in the market, which are typically associated with unpredictable pharmacokinetics.^[^ ^8^ ^]^ In addition, dosage restrictions due nephrotoxicity issues make polymyxins less efficacious in vivo.^[^ ^9^ ^]^ To reduce toxicity and enhance the antimicrobial performance of PMB, various efforts have been made in two main directions. One direction has been focused on the synthesis of new PMB derivatives based on medicinal chemistry principles,^[^ ^6^, ^10^ ^]^ while the other has been centered around the development of new PMB formulations through nanomedicine‐based approaches,^[^ ^11^ ^]^ such as nanoparticles,^[^ ^12^ ^]^ liposomes,^[^ ^13^ ^]^ and hydrogels,^[^ ^14^ ^]^ for the controlled release of PMB directly at the infection sites.

Hydrogels have gained extensive interest for the controlled delivery of therapeutics.^[^ ^15^ ^]^ Owing to their tunable mechanical properties, hydrogels can be either injected or implanted at the desired sites, acting as reservoirs for the localized and sustained release of drugs. Their high degree of water content makes them particularly attractive as wound care materials. Compared to other materials, hydrogels can prevent the loss of body fluids and keep the wound surface hydrated, support the release of therapeutics and promote the healing process, as well as act as barriers against bacterial infections.^[^ ^16^ ^]^ The use of peptides as building blocks for hydrogel fabrication has been increasingly appealing due to their inherent biocompatibility and biodegradability.^[^ ^17^ ^]^ In addition, through rational design of the peptide sequences, biological and environmentally responsive functionalities can be incorporated into the hydrogels, enabling their diverse biomedical applications.^[^ ^18^ ^]^ Several peptide‐based hydrogels, such as *β*‐hairpin and multidomain peptide hydrogels, initially exploited for applications in regenerative medicine, have now been modified and assessed for their antimicrobial activities.^[^ ^19^ ^]^

Since peptides possess chemical groups sensitive to thermal, enzymatic, and chemical conditions, their gelation via physical cross‐linking has been more frequently utilized rather than covalent cross‐linking which typically requires additional chemical modifications. For example, multivalent ions, such as PO_4_^3−^ and Ca^2+^, have been shown to shield the opposite charges of peptides and trigger their self‐assembly into hydrogel networks.^[^ ^20^ ^]^ Similarly, anionic drugs such as heparin and suramin have also been used to promote the physical cross‐linking of cationic multidomain peptide nanofibers into self‐supporting hydrogels.^[^ ^21^ ^]^ In this context, the cationic PMB was investigated here as a potential ionic cross‐linker and trigger of the supramolecular gelation of peptides with opposite charge. Self‐assembling peptide amphiphiles (PAs) containing negatively charged carboxyl groups were designed and their PMB‐triggered gelation was examined from the molecular to the macroscopic level. The capacity of the PMB‐triggered PA hydrogel as an effective formulation for sustained PMB delivery was assessed by determining the release profile of PMB from the hydrogel. Their antibacterial performance was evaluated both in vitro by disk transfer and diffusion method and in vivo on a *Galleria mellonella* burn wound infection model. Moreover, proof‐of‐concept experiments were performed to examine the capacity of the hydrogel to incorporate additional antibiotics for combined antimicrobial therapy. The PMB‐triggered PA hydrogel, obtained by molecular self‐assembly in mild environmental conditions, provides a simple and versatile formulation for the localized and sustained delivery of PMB.

## 2 Results and Discussion

### 2.1 Molecular Design and Assembly

**Figure 1:**
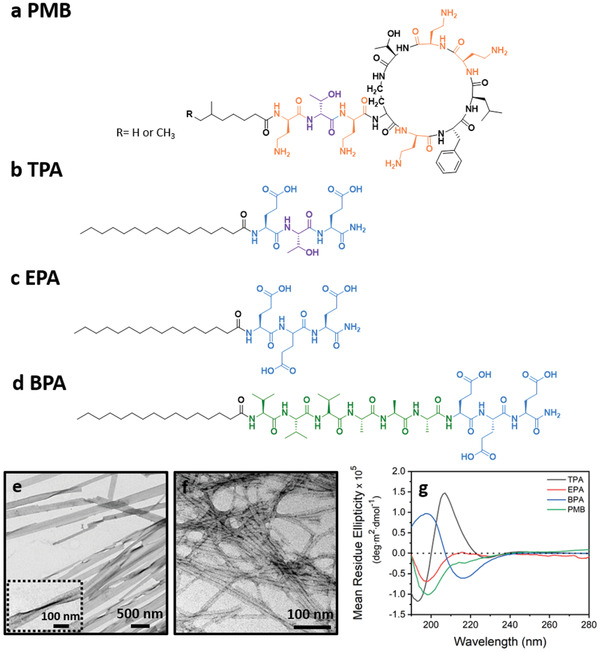
Molecular design and assembly of the peptide amphiphiles (PAs). Chemical structures of a) polymyxin B and the rationally designed b) TPA, c) EPA, and d) BPA. Representative TEM images showing e) lamellar nanofibers for TPA and f) cylindrical nanofibers for BPA. g) Circular dichroism spectra displaying *β*‐sheet secondary structure for the TPA and BPA nanofibers, and random coil conformation for the EPA and PMB solutions.

As displayed in **Figure** **1a**, the PMB molecule contains five primary amines in its structure, which can establish electrostatic interactions with molecules or assemblies of opposite charges and eventually induce gelation. To investigate the possibility and mechanism of the PMB‐triggered gelation of peptide assemblies, three variations of PAs were designed. All the three PAs contain glutamic acid residues in their sequences, displaying ionizable carboxylic acid groups on their side chains. Among them, the threonine (T)‐containng PA (TPA) has a tripeptide headgroup, glutamic acid–threonine–glutamic acid (Figure 1b), which was designed to form paired interactions with the tripeptide sequence, diaminobutyric acid–threonine–diaminobutyric acid, in the linear tail of PMB. To elucidate the role of hydrogen bonding between the threonine residues, a glutamic acid (E)containing PA (EPA) was designed by substituting the middle threonine in TPA by glutamic acid (Figure 1c). Moreover, a beta‐sheet forming PA (BPA, Figure 1d), known to assemble into nanofiber structures and containing a segment of six amino acids (VVVAAA) between the hydrophobic alkyl tail and the hydrophilic peptide headgroup of EPA, was used here to reveal the influence of PA secondary structure on its eventual gelation triggered by PMB. All the three PAs were successfully synthesized and purified, with their mass and purity being confirmed by electrospray ionization mass spectrometry (ESI‐MS) and reverse‐phase high‐performance liquid chromatography, respectively (Figures S1–S3, Supporting Information).

Using Nile red, a solvatochromic fluorescent dye that emits increased fluorescence intensity with pronounced blueshift when encapsulated into the hydrophobic region of PA assemblies,^[^ ^22^ ^]^ the critical aggregation concentration (CAC) was determined to be 0.02 and 0.002 mg mL^−1^ for TPA and BPA, respectively (Figure S5, Supporting Information). However, no detectable CAC was obtained for EPA in a concentration range up to 10 mg mL^−1^. When dissolved in water at 0.2 mg mL^−1^ (above their CAC), TPA assembled into bundled lamellar nanofibers with diameters of ≈180 nm (Figure 1e), while BPA formed cylindrical nanofibers with diameters around 10 nm as previously reported (Figure 1f).^[^ ^23^ ^]^ To further reveal the internal structure of the assembled nanofibers, circular dichroism (CD) characterization was performed. As displayed in the CD spectra (Figure 1g), a maximum at 207 nm and a minimum at 227 nm were observed for the TPA nanofibers, which differ from the canonical *β*‐sheet conformation observed for the BPA nanofibers, exhibiting a maximum at 195 nm and minimum at 216 nm.^[^ ^24^ ^]^ The redshifted *β*‐sheet signal was previously reported to be associated with increased twisting or disorder in the *β*‐sheet hydrogen bonding.^[^ ^25^ ^]^ Consistent with this finding, slightly twisted TPA nanofibers could be observed by transmission electron microscopy (TEM) imaging (Figure 1e inset), which was also considered to constrain the unlimited lateral growth of these lamellar nanofiber.^[^ ^26^ ^]^ By contrast, EPA displayed a random coil conformation (Figure 1g) and no well‐defined nanostructures could be found under TEM, probably due to the strong repulsive forces among the negatively charged headgroups, preventing its assembly into stable structures. Thereby, only the lamellar TPA nanofibers and cylindrical BPA nanofibers were further investigated here for their ability to form hydrogels in the presence of PMB.

### 2.2 PMB‐Triggered Gelation

**Figure 2:**
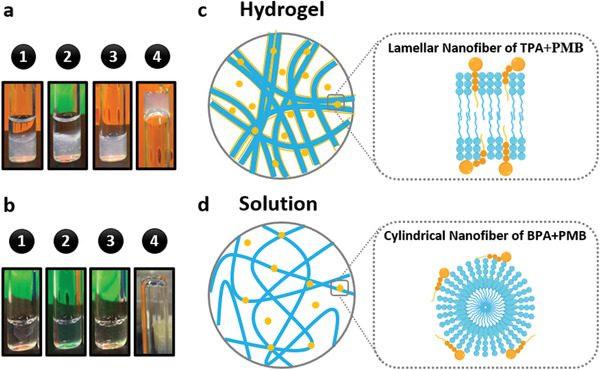
Polymyxin B (PMB)‐triggered gelation of PAs. a1) Hydrogel was formed by adding an equal volume of 2 mg mL^−1^ PMB solution into 20 mg mL^−1^ TPA solution and a2) remained stable after 24 h. a3) TPA solution alone, before adding PMB solution, and a4) reverse vial test demonstrating its high viscosity. b1) Membrane was formed by adding an equal volume of 2 mg mL^−1^ PMB solution into 20 mg mL^−1^ BPA solution but b2) disappeared after 24 h. b3) BPA solution alone, before adding PMB solution, and b4) reverse vial test demonstrating its high fluidity. Proposed interactions between the PMB molecules with c) lamellar TPA nanofibers and d) cylindrical BPA nanofibers.

With the addition of 2 mg mL^−1^ PMB solution into an equal volume of 20 mg mL^−1^ TPA solution, a translucent and stable hydrogel was formed (**Figure** **2a1**,a2). However, when the PMB and BPA solutions were similarly combined, only an opaque thin membrane was observed (Figure 2b1), which was not stable and disappeared after overnight incubation at 37 °C (Figure 2b2). Given that the main difference between the two negatively charged PA nanofibers is their molecular organization, the PMB‐triggered gelation of TPA could be attributable to the comparatively loose molecular packing inside the lamellar TPA nanofibers. As depicted in Figure 2c, the less cohesive molecular organization of TPA in the lamellar nanofibers is expected to allow PMB to intercalate into the assemblies. Accordingly, the hydrophobic tails and hydroxyl side groups of threonine in both the TPA and PMB molecules could be brought into close contact, favoring the formation of hydrophobic interactions and hydrogen bonding. As a consequence, the increased hydrogen bonding and hydrophobic forces between the TPA and PMB molecules could stabilize the weakly formed ionic linkage between the carboxyl groups of TPA and the amino groups of PMB, enhancing the internal cohesion of the assemblies. In this configuration, the amino groups in the PMB ring could further form electrostatic interactions with other TPA nanofibers, promoting the cross‐linking of TPA nanofibers and leading to the formation of stable hydrogels (Figure 2c). By contrast, the *β*‐sheet forming domain of BPA is known to promote strong hydrogen bonding among the BPA molecules,^[^ ^27^ ^]^ enabling their radial organization and growth into cylindrical nanofibers with more tightly packed BPA molecules, which may prevent the insertion of the lipid tail of PMB into the hydrophobic core of BPA nanofibers (Figure 2d). As a result, the interactions between BPA and PMB could merely occur at the surface of the BPA nanofibers via electrostatic interactions. The lack of additional interactions, such as hydrogen bonding and hydrophobic interactions between the BPA nanofibers and PMB molecules, results in the formation of unstable membranes.

Apart from the different molecular packing within the nanofibers, another noted difference between TPA and BPA is the viscosity of their own aqueous solutions before the addition of PMB solution. The difference in viscosity is expected to affect the mixing kinetics when PMB is added and consequently leading to different modes of interaction. Compared to the BPA solution with the same concentration, TPA solution was more viscous, with reduced fluidity as seen in the inverted‐vial test (Figure 2a4). Therefore, various negatively charged polymers known to generate high viscous solutions were selected to investigate whether the PMB‐triggered gelation was viscosity‐dependent. These polymers include alginate, hyaluronic acid, and polyacrylic acid. Upon interaction with PMB, opaque interfaces were observed for alginate and polyacrylic acid but disappeared after overnight incubation at 37 °C, while no evident changes were observed for hyaluronic acid (Table S1, Supporting Information). Overall, none of these highly viscous solutions of anionic polymers were able to form stable hydrogels with the addition of PMB.

Collectively, the presence of negative charge and high viscosity were not sufficient for the PMB‐triggered gelation. The rationally designed TPA, which assembled into lamellar nanofibers with relatively loose molecular packing, is likely to be determinant for permitting intercalation of PMB within the lamellar nanofibers, enhancing their internal cohesion and promoting their cross‐linking into stable hydrogels.

### 2.3 Nanofibrous Hydrogel Network

**Figure 3:**
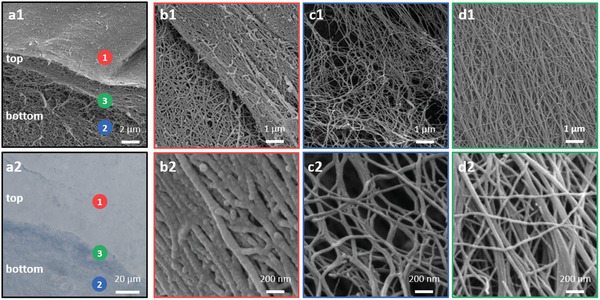
Nanofibrous network of the PMB‐triggered PA hydrogel. a1) Representative SEM image of the PMB‐triggered PA hydrogel, showing distinct fibrous network at the surface of the top layer (labeled as 1 in a1), b1) and b2) are images at higher magnification); bottom layer (labeled as 2 in a1), c1) and c2) are images at higher magnification); and interface near the top and bottom layer (labeled as 3 in a1), d1) and d2) are images at higher magnification). a2) Optical microscopy image of the toluidine blue‐stained hydrogel, exhibiting two distinct top and bottom layers as observed in a1)

The PMB‐triggered PA hydrogel was observed to have an opaque thin top layer and a more transparent thick bottom layer (Figure 2a2). To elucidate the difference in the two layers, scanning electron microscopy (SEM) was used to examine their nanoscale structure (**Figure** **3a1**). As shown in Figure 3b1,b2, well‐packed thick fibrous nanostructures were observed for the surface of the top layer. By contrast, more porous and thinner nanofibers were visualized for the bottom layer (Figure 3c1,c2). Coexistence of both thick and thin nanofibers could be found at the interface between the top and bottom layer of the hydrogel (Figure 3d1,d2). To further reveal the composition of the two distinct layers, the hydrogel was sectioned by cryostat microtome and stained with toluidine blue, a basic dye used in histology for staining acidic macromolecules in tissues.^[^ ^28^ ^]^ Compared to the top layer, the bottom layer of the hydrogel exhibited a more intense blue color (Figure 3a2), indicating less PMB in the bottom layer than in the top layer of the hydrogel.

Taken together, the PMB‐triggered PA hydrogel was demonstrated to have two distinct layers with respect to the nanofiber network. The top layer is a dense contact layer of PMB molecules and TPA lamellar nanofibers formed by charge complexation, acting as a diffusion barrier. As a result, more PMB molecules accumulate in the top layer, so that the toluidine blue could barely stain the top layer. By contrast, the bottom layer exhibits intense blue color with the toluidine blue staining, containing less diffused PMB molecules and displaying a relatively less cross‐linked nanofiber network.

### 2.4 Optimized Hydrogel Composition

**Figure 4:**
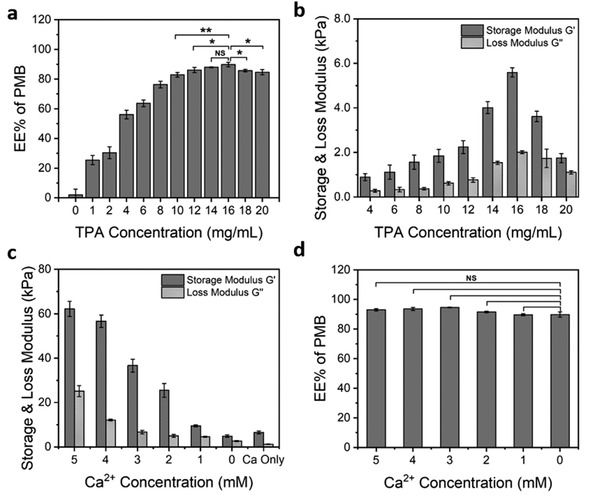
Tunable PMB encapsulation efficiency (EE%) and mechanical stiffness of the PMB‐triggered PA hydrogels: a,d) effect of TPA and calcium ion concentration on the PMB EE% and b,c) mechanical stiffness of the PMB‐triggered PA hydrogels. Data are given as mean ± SD (*n* = 3), with *p ≤ 0.05, **p ≤ 0.01.

To determine the optimum proportion of PMB and TPA used for the hydrogel preparation, a fixed concentration of PMB solution at 2 mg mL^−1^ was used to trigger the gelation of TPA solution with varying concentrations. Quantified by the fluorescamine assay,^[^ ^29^ ^]^ the encapsulation efficiency (EE%) of PMB in the hydrogels was found to increase when the concentration of TPA solution increased from 4 to 16 mg mL^−1^ (**Figure** **4a**). A maximum encapsulation efficiency of PMB was achieved at 89.78% in the hydrogel prepared with 16 mg mL^−1^ TPA solution. However, further increase in the TPA concentration resulted in a slightly decreased EE% of PMB in the hydrogels (Figure 4a).

Given that these hydrogels contain only two components, PMB and TPA, their mechanical properties should also be affected by changing the proportion of PMB and TPA incorporated. As such, oscillatory rheological measurements were performed on these PMB‐triggered PA hydrogels. Amplitude sweeps demonstrated that these hydrogels could maintain their linear viscoelastic region with up to 1% oscillation strain at frequency of 1 Hz (Figure S7, Supporting Information). Therefore, frequency sweeps were performed at 0.1% strain with change in frequency. As shown in Figure 4b, the change in the mechanical stiffness of these hydrogels followed the same trend as the change in the PMB EE%. A steady increase in both storage (*G′*) and loss (*Gʺ*) moduli was observed with increasing TPA concentration up to 16 mg mL^−1^, after which a decrease in both the *G′* and *Gʺ* was observed with further increase in TPA concentration.

These results are notable as the developed formulation enables high EE% and enhanced mechanical properties. Overall, equal volumes of 2 mg mL^−1^ PMB solution and 16 mg mL^−1^ TPA solution provided the optimized composition in the hydrogel, with the maximum EE% of PMB and the highest mechanical stiffness.

### 2.5 Tunable Mechanical Properties

Antibacterial hydrogels intended for local delivery require adaptable mechanical properties to facilitate their application at different tissue sites. For example, when treating fracture or implant‐related bone infections, a higher stiffness is typically desired to provide mechanical stability.^[^ ^30^ ^]^ Therefore, the possibility to regulate the mechanical properties of the PMB‐triggered PA hydrogels was exploited here to expand their potential applications. Even though tunable stiffness of the PMB‐triggered PA hydrogel could be obtained by adjusting the concentration of TPA, the variation range is narrow with the highest *G′* obtained under 10 kPa (Figure 4b). To further increase their stiffness, calcium chloride (CaCl_2_) solution was used to dissolve PMB for the hydrogel preparation, instead of pure water, because calcium ions were previously used for shielding the negative charges of self‐assembling peptides, promoting intra‐ and intermolecular cross‐linking and allowing the formation of nanofibers and self‐supporting hydrogels.^[^ ^20b^ ^]^ As expected, a 5 × 10^−3^ m CaCl_2_ solution, with equivalent number of positive charges as in the 2 mg mL^−1^ PMB solution, could also trigger TPA gelation with the hydrogel exhibiting a slightly higher *G′* (6526 ± 697 Pa) but lower *Gʺ* (1219 ± 149 Pa) than the PMB‐triggered PA hydrogel with *G′* at 5429 ± 432 Pa and *Gʺ* at 2011 ± 124 Pa (Figure 4c). In addition, increasing the calcium ionic strength of PMB solution from 0 to 5 × 10^−3^ m resulted in an approximately tenfold increase in the mechanical stiffness of the formed hydrogels (Figure 4c). Meanwhile, no significant difference was observed for the EE% of PMB in the formed hydrogels with increasing concentration of calcium ions (Figure 4d). These results suggest that the addition of calcium ions can promote further cross‐linking of the hydrogel network without interfering with the interactions between PMB and TPA nanofibers. The tunable mechanical properties of these hydrogels, adjusted by the amount of calcium ions added into the PMB solution for hydrogel preparation, reveal their potential as implantable antimicrobial biomaterials.

To gain further insight into the effect of the hydrogels’ mechanical properties and PMB EE% on their antibiotic release and antibacterial activities, three representative hydrogels with varying stiffnesses and EE% of PMB were selected to perform the experiments described in the following sections. Among which, the PMB Gel is referred to the hydrogel triggered by 2 mg mL^−1^ PMB solution; the PMB+Ca Gel referred to hydrogel triggered by 2 mg mL^−1^ PMB in 5 × 10^−3^ m CaCl_2_ solution; and the Ca Gel referred to hydrogel triggered by 5 × 10^−3^ m CaCl_2_ solution.

### 2.6 Sustained Polymyxin B Release

**Figure 5:**
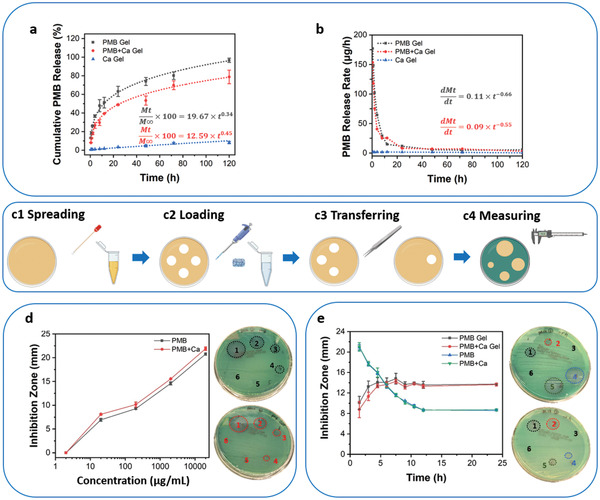
Sustained PMB release and prolonged bacterial inhibition activity against *P. aeruginosa* PA14 of the PMB‐triggered PA hydrogels. a) Cumulative PMB release profile of three representative hydrogels in 1 mL PBS over a 5‐day period, with the optimum fitting curves and equations modeled. b) PMB release rates of the three hydrogels determined as the first derivative of cumulative release displayed in a). c) Schematic illustration of the modified disk transfer and diffusion method, including c1) spreading the bacterial inoculum over the agar plates; c2) loading the PMB hydrogels or solutions onto the blank disks (6 mm) placed on the agar plates; c3) transferring the disks onto the next agar plate after 1.5 h diffusion; c4) measuring the inhibition zones displayed on the agar plates after overnight incubation at 37 °C. d) Change of inhibition zone in response to the different concentrations of PMB, with pictured agar plates showing susceptibility of the *P. aeruginosa* PA14 to PMB solution (top) and PMB+Ca solution (bottom) presented in the dose response assay (1 ‐ 20 000 µg mL^−1^, 2 ‐ 2000 µg mL^−1^, 3 ‐ 200 µg mL^−1^, 4 ‐ 20 µg mL^−1^, 5 ‐ 2 µg mL^−1^, 6 ‐ PBS). e) Change of the inhibition zone over time for the different formulations of PMB, with two representative agar plates at time 1.5 h (top) and 12 h (bottom) presented in the disk transfer and diffusion assay (1 ‐ PMB Gel, 2 ‐ PMB+Ca Gel, 3 ‐ Ca Gel, 4 ‐ PMB Solution, 5 ‐ PMB+Ca Solution, and 6 ‐ PBS). Data are given as mean ± SD (*n* = 3).

The antibiotic release behavior of the PMB‐triggered PA hydrogels was investigated in phosphate‐buffered saline (PBS), which was placed over the top of the hydrogels. As demonstrated in **Figure** **5a**, the PMB‐containing hydrogels (PMB Gel and PMB+Ca Gel) displayed sustained PMB release, with up to 96.6 ± 2.3% release achieved for the PMB Gel over a 5‐day period, while relatively less release was observed for the PMB+Ca Gel (78.7 ± 7.3%). The release profile of PMB was then modeled and Equation (1) (*R* ^2^ = 0.97) and Equation (2) (*R* ^2^ = 0.99) were obtained for the PMB Gel and PMB+Ca Gel, respectively, in which *t* is the release time, *Mt* is the cumulative amount of PMB released at time *t*, and *M*∞ is the total PMB amount used to prepare the hydrogels

(1) $$\frac{Mt}{M\infty }\times 100\%=19.67\times t^{0.34}$$

(2) $$\frac{Mt}{M\infty }\times 100\%=12.59\times t^{0.45}$$

The two equations conform to the Peppas equation, *Mt*/*M*∞  =  *kt^n^*, in which *k* is the constant on the structure and geometric properties of the drug loading matrix, and *n* is the release index, with *n* = 0.5 suggesting the diffusion‐dependent release kinetics, *n* = 1 indicating the erosion‐dependent release kinetics, and 0.5 < *n* < 1 signifying diffusion and erosion codependent release kinetics.^[^ ^31^ ^]^ In Equations (1) and (2), *n* ≈ 0.5, suggesting that the PMB release kinetics is diffusion‐dependent. These values of *n* are not exactly 0.5 because the two extrema, 0.5 and 1, only apply to the slab matrix. For matrixes with different geometries, such as spherical and cylindrical, values of the two extrema are different.^[^ ^31^, ^32^ ^]^ First derivative of the release profiles showed that the release rates for both PMB‐containing PA hydrogels were initially faster, but then tapered after 8 h to a nearly constant release rate lower than 10 µg h^−1^ (Figure 5b). However, compared to the PMB Gel, the final release amount and release rate of PMB from the PMB+Ca Gel were relatively lower (Figure 5a,b). These may be caused by the decelerated diffusion process of PMB from the PMB+Ca Gel, as enhanced mechanical properties were observed for the PMB+Ca Gel than the PMB Gel, which suggested a more tightly packed network formed in the PMB+Ca Gel with the addition of calcium ions. Overall, sustained PMB release from the PMB‐triggered PA hydrogels via a diffusion‐dependent kinetic was observed. The final release amount and release rate of PMB from the hydrogels could be altered with the addition of calcium ions, making the dosage optimization possible to meet different clinical demands.

### 2.7 Prolonged Antimicrobial Activity

Compared to the soluble formulation of PMB, the PMB‐triggered PA hydrogels were hypothesized to possess prolonged antimicrobial activity since sustained PMB release over a 5‐day period was demonstrated. Since the PMB is a proven bactericide with rapid membrane disruption ability,^[^ ^33^ ^]^ the prolonged in vitro antimicrobial activity of the PMB‐triggered PA hydrogels was examined by a previously reported disk transfer and diffusion method,^[^ ^34^ ^]^ rather than the colony count assay typically used to assess the capacity for inhibition of bacterial growth.^[^ ^19c^ ^]^ As illustrated in Figure 5c, disks loaded with either PMB solution or hydrogel (containing the same amount of PMB as in the solution) were transferred every 1.5 h onto the next bacterial‐suspension‐swabbed agar plate. After overnight culturing, clear bacteria‐free zones, also known as inhibition zones, could be observed from the disk loading different PMB formulations.

Gram‐negative *Pseudomonas aeruginosa* PA14 was chosen to perform the experiment and its susceptibility to PMB diffused from the disk was first verified by the dose response assay. The minimum inhibitory concentration (MIC) of PMB against PA14, determined by broth microtiter dilution, is 0.5 µg mL^−1^.^[^ ^35^ ^]^ However, no inhibitory effect was observed from the disk loading 2 µg mL^−1^ PMB solution (Figure 5d), which is probably due to the limited amount of PMB diffused into the agar plate over 1.5 h. Inhibition zones started to appear around disk loading 20 µg mL^−1^ PMB solution. Above this concentration, the diameter of the formed inhibition zone was shown to be positively correlated with the logarithm of PMB concentration (Figure 5d). The greater the PMB concentration loaded into the disk, the higher the amount of PMB could diffuse into the agar over 1.5 h, and therefore the larger the inhibitory zone against *P. aeruginosa* PA14.

In the disk transfer and diffusion experiment, a gradual decline in the inhibitory effect against *P. aeruginosa* PA14 was observed for disks loaded with solutions containing PMB in the free form, with only limited and unchanged antibacterial effect being observed after 12 h (Figure 5e and Figure S9, Supporting Information). By contrast, increasing inhibitory effect was observed for disks loaded with the PMB‐triggered PA hydrogels in the first 4.5 h, and since then a significant level of inhibitory effect was maintained up to 24 h (Figure 5e). These results suggested that the PMB‐triggered PA hydrogels could provide sustained PMB release and subsequently prolong the antimicrobial activity against *P. aeruginosa* PA14. The concentration of PMB released at each time point was always above its MIC (0.5 µg mL^−1^),^[^ ^35^ ^]^ therefore minimizing the risk for inducing microbial resistance.^[^ ^36^ ^]^ The addition of calcium ions to the soluble PMB formulation did not significantly affect its antimicrobial profile. Despite slightly weakened antimicrobial activities were observed when the calcium ions were added into the hydrogel (Figure 5e), probably due to the reduced PMB release from the hydrogel containing calcium ions, the observed difference is not statistically significant. In addition, the PMB‐triggered PA hydrogels were also proven to have extended antimicrobial effects against the Gram‐negative *Escherichia coli* (Figure S10, Supporting Information) and *Acinetobacter baumannii* (Figure S11, Supporting Information). The sustained PMB release and prolonged antimicrobial activities of the PMB‐triggered PA hydrogels indicated their potential to act as localized reservoirs for the delivery of PMB and treatment of persistent infections caused by Gram‐negative bacteria.

### 2.8 Effective In Vivo Antimicrobial Potency

**Figure 6:**
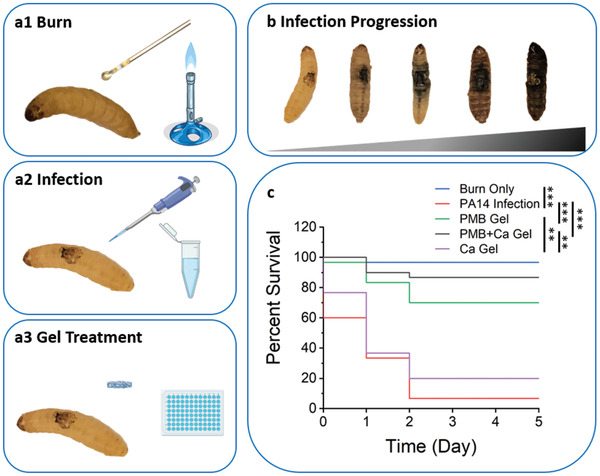
Antimicrobial efficiency of the PMB‐triggered PA hydrogels assessed on the *G. mellonella* burn wound infection model. a) Schematic illustration of the treatment procedure on the *G. mellonella* model, including a1) burning the middle part of *Galleria* back using a metal device heated by the Bunsen burner; a2) infecting the burn wound with 10 µL of overnight cultured *P. aeruginosa* PA14 bacterial inoculum (1:10 dilution); a3) treating the infected burn wound with the various hydrogels coated directly onto the burn area. b) Illustration of the infection progression on the *G. mellonella* model over time, with representative images showing the gradual changing stage toward melanization and loss of motility of the *Galleria* with the bacterial dissemination. c) Survival curves for *P. aeruginosa* PA14 infected *Galleria* with the PMB‐triggered PA hydrogel treatment. Triplicate set of ten *Galleria* were tested in each group. Statistical significance of differences was determined based on the pooled dada using a Logrank (Mantel–Cox) test with *p ≤ 0.05, **p ≤ 0.01, and ***p ≤ 0.001.

To validate the in vivo antimicrobial efficiency of the PMB‐triggered PA hydrogels, experiments were performed on an invertebrate *G. mellonella* model, as an alternative to mammalian models, such as mice and rabbits. Compared to the mammalian models, the *G. mellonella* model is more cost‐effective and less challenging in ethics, requires shorter experimental cycle, while providing larger volume of data for higher power statistical analysis.^[^ ^37^ ^]^ It has become an increasingly popular nonmammalian model for studying bacterial infections and screening antimicrobial drugs.^[^ ^38^ ^]^ Previously, a self‐assembling peptide hydrogel (NapFFKK—OH) was examined on the *G. mellonella* infection model through injection for investigating its in vivo toxicity and antimicrobial activity.^[^ ^39^ ^]^ To facilitate additional administration routes, a *G. mellonella* burn wound infection model was established recently by us and collaborators (**Figure** **6a**,b), which could closely match etiological manifestation of burn wound infections in humans and other mammalian models.^[^ ^40^ ^]^ After having confirmed the safety profile of TPA and PMB in *Galleria*, through direct injection of solutions at concentrations used for the hydrogel preparation (20 mg mL^−1^ and 2 mg mL^−1^, respectively, Figure S12, Supporting Information), the PMB‐triggered PA hydrogels were applied directly onto the infected burn wound of *Galleria* (Figure S13C, Supporting Information) for evaluating their in vivo antimicrobial efficacy.

As shown in Figure 6c, the burn protocol for wound creation was well‐tolerated by *Galleria*, as more than 95% of the larvae in the control (Burn Only) group were alive after 5 days at 37 °C. Longer‐time observation was avoided because the larvae began to cocoon after 5 days. A highly pathogenic *P. aeruginosa* strain PA14, originally isolated from a human burn wound patient, was chosen for infecting the wounded *Galleria*.^[^ ^41^ ^]^ The infection led to a high level of mortality, with only 7% survival observed after 5 days for the PA14 Infection group. After applying the PMB‐triggered PA hydrogels, significant reduction in mortality was observed in both the PMB Gel and PMB+Ca Gel treated groups. More interestingly, the PMB+Ca Gel was observed to be slightly more effective than the PMB Gel, in terms of promoting the survival rate of the larvae, despite exhibiting comparatively lower cumulative PMB release amount. Approximately 87% of the larvae were observed to survive after 5 days when treated with the PMB+Ca Gel, while only around 70% survival was observed in the PMB Gel treated group. The increased survival with the PMB+Ca Gel treatment may be due to its enhanced mechanical properties compared to the PMB Gel, which enables the hydrogel to remain in place and promotes better sealing of the wound. Surprisingly, the Ca Gel treatment group, which served as the negative control group, also promoted 20% survival after 5 days. The slightly improved survival of *Galleria* is probably due to the beneficial effect from the enhanced wound hydration provided by the Ca Gel treatment, as severe fluid loss has been reported to be partly responsible for mortality in the established *G. mellonella* burn wound infection model.^[^ ^40^ ^]^ Altogether, the PMB‐triggered PA hydrogels were demonstrated to be effective in vivo for treating Gram‐negative *P. aeruginosa* PA14 infection on a *G. mellonella* burn wound infection model, by significantly reducing the mortality from 93% to 13%.

### 2.9 Combined Antimicrobial Therapy

**Figure 7:**
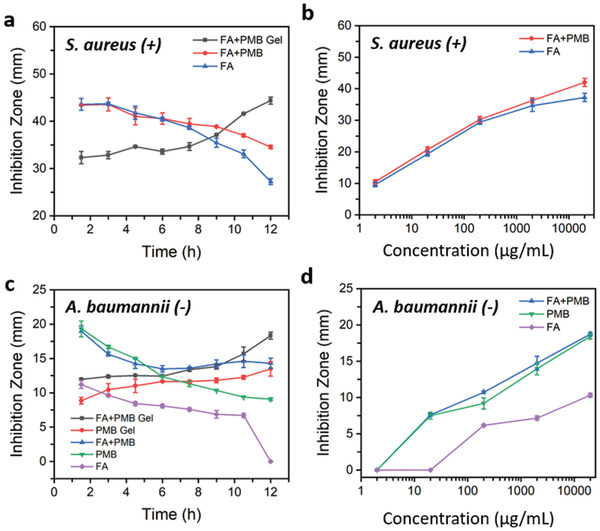
Bacterial inhibition activities of the combined fusidic acid and polymyxin B (FA+PMB) formulations against Gram‐positive *S. aureus* and Gram‐negative *A. baumannii*. a) Change of the inhibition zones over time for FA+PMB Gel, FA+PMB Solution, and FA Solution, displayed on the agar plates in the disk transfer and diffusion test against *S. aureus*; PMB Gel and PMB Solution displayed no bacterial inhibition activity against *S. aureus* and therefore no inhibition zones were observed. b) Inhibition zones for various concentrations of FA+PMB Solution and FA Solution, displayed on the agar plates in the susceptibility test against *S. aureus*. c) Change of the inhibition zones over time for FA+PMB Gel, PMB Gel, FA+PMB Solution, PMB Solution. and FA Solution, displayed on the agar plates in the disk transfer and diffusion test against *A. baumannii*. d) Inhibition zones for various concentrations of FA+PMB Solution, PMB Solution, and FA Solution, displayed on the agar plates in the susceptibility test against *A. baumannii*. Data are given as mean ± SD (*n* = 3).

Taking advantage of the nanofibrous and porous architecture of the PMB‐triggered PA hydrogel, the incorporation of additional antibiotics into the hydrogel network was considered for the development of combined antimicrobial therapy. Given that PMB is only effective in killing Gram‐negative bacteria, fusidic acid (FA), an antibiotic that is effective in killing Gram‐positive bacteria,^[^ ^42^ ^]^ was chosen as a model for evaluating the possible therapeutic combination. To prepare hydrogels containing the two antibiotics, FA solution (2 mg mL^−1^) was first prepared and used to dissolve the TPA at concentration of 16 mg mL^−1^. Upon addition of the 2 mg mL^−1^ PMB solution, FA+PMB Gel was formed, indicating that the PMB‐triggered hydrogel assembly was not affected by the incorporation of FA. As shown in **Figure** **7a**, the incorporated FA could be effectively released from the FA+PMB Gel and remained active against Gram‐positive *Staphylococcus aureus*. By contrast, no such activity was observed for the PMB Gel. In addition, compared to the FA and FA+PMB solutions, the FA+PMB Gel also exhibited sustained antimicrobial activity against *S. aureus*, with increasing inhibition zones being observed over time (Figure 7a). However, FA+PMB solution only displayed higher antimicrobial potency than the FA solution after 8 h in the disk transfer and diffusion test (Figure 7a) and exhibited no significant difference in the susceptibility test (Figure 7b). Compared to FA alone, whether the synergistic effect against *S. aureus* exists between FA and PMB still needs to be further investigated by checkerboard assay. Nevertheless, the incorporation of FA endows the PMB‐triggered PA hydrogel with additional ability in killing Gram‐positive bacteria.

The combination of FA with colistin (polymyxin E) was shown to have a potent synergistic interaction against MDR Gram‐negative *A. baumannii* pathogens, including those with resistance to polymyxins.^[^ ^43^ ^]^ Extending on this, the killing potency of the FA+PMB Gel against *A. baumannii* was also examined here. As shown in Figure 7c, inhibition zones with increasing diameter were observed for both the FA+PMB Gel and the PMB Gel during the whole 12 h period examined, indicating that the antibiotics were sustained released from the hydrogel and the released antibiotics were still active against *A. baumannii*. More importantly, the FA+PMB Gel was shown to be more effective against *A. baumannii* than the PMB Gel, as larger inhibition zones were observed for the FA+PMB Gel. In addition, soluble FA+PMB formulations also exhibited enhanced antimicrobial efficiency compared to the FA solution in both the disk transfer and diffusion test (Figure 7c), as well as the susceptibility test (Figure 7d). However, FA+PMB solution only displayed enhanced antimicrobial activities after 6 h compared to the PMB solution (Figure 7c) and no significant difference was found between them in the susceptibility test (Figure 7d). Even though the release mechanism of FA from the hydrogel and the synergistic effect of FA with PMB examined by the checkerboard assay still require further investigation, it appears that the FA+PMB Gel could effectively release the two antibiotics and promote enhanced antimicrobial potency.

Taken together, the above results demonstrated the possibilities of the PMB‐triggered PA hydrogel, with incorporation of FA, to serve as combined antimicrobial therapy for the treatment of infections caused by either multiple bacterium (Gram‐positive and Gram‐negative) or by MDR Gram‐negative bacteria.

## 3 Conclusion

In this work, the decade‐old PMB antibiotic was repurposed into a more effective hydrogel formulation to tackle the evolving clinical and market demand of antimicrobial therapies for treating persistent and life‐threatening infections caused by Gram‐negative bacteria. The formulation is based on the ability of PMB to act as ionic cross‐linker of aqueous solutions containing PA molecules, designed to self‐assemble into lamellar *β*‐sheet nanofibers, and trigger their conversion into a robust hydrogels. By controlling the amount of divalent calcium ions in the formulation, PMB‐triggered PA hydrogels with tunable mechanical properties could be obtained, enabling their application as local depots for sustained PMB release in a range of infection scenarios, such as skin wounds or fracture fixation systems. The PMB‐triggered PA hydrogels enabled extended PMB release via diffusion‐dependent kinetics and prolonged antimicrobial activities against Gram‐negative bacteria, when compared to the soluble PMB formulation. Extended‐release formulations for the local delivery of antimicrobial drugs are advantageous as they maximize their therapeutic effect via high local concentrations, while minimizing systemic exposure and risk for antibiotic resistance. Moreover, these formulations are particularly beneficial for treating persistent infections and improve patient compliance, when compared to conventional systemic delivery of PMB (e.g., intravenous administration) that requires three daily dosages for 1–2 weeks. In fact, the localized release of active PMB from the hydrogels resulted in dramatically reduced mortality in vivo on a *G. mellonella* burn wound infection model (13% compared to the 93% mortality caused by *P. aeruginosa* PA14 infection without treatment), demonstrating the potential of the PMB‐triggered PA hydrogel for treating chronic wound infections caused by Gram‐negative superbugs, including MDR bacteria. Furthermore, the successful incorporation of additional antibiotics (e.g., fusidic acid) into the porous network of the PMB‐triggered PA hydrogel displayed complementary antimicrobial activity against Gram‐positive bacteria and also enhanced effect against Gram‐negative bacteria, envisioning their potential for combined antimicrobial therapy. We believe that these findings provide insights into the rational design and development of simple and versatile antibiotic formulations for improved local treatment of MDR bacterial infections.

## 4 Experimental Section

### Materials

Fluorenylmethyloxycarbonyl (Fmoc)‐amino acids and 4‐methylbenzhydrylamine (MBHA) rink amide resin were acquired from Novabiochem (UK). 1‐hydroxybenzotriazole (HOBt) was purchased from Carbosynth (UK). *N*,*Nʹ*‐diisopropylcarbodiimide, piperidine, dimethylformamide (DMF), dichloromethane, trifluoroacetic acid (TFA), triisopropylsilane (TIS), diethyl ether, acetonitrile (ACN), palmitic acid, hydrochloric acid, ammonium hydroxide (NH_4_OH), CaCl_2_, Nile red, fluorescamine, paraformaldehyde, toluidine blue, Kaiser Test Kit, alginic acid sodium salt form (medium viscosity), and poly(acrylic acid) (average *M*_w_ ≈ 1 250 000 Da) were purchased from Sigma‐Aldrich (UK) and used without further purification. Hyaluronic acid (1.5 MDa) was obtained from Lifecore Biomedical (USA). Uranyl acetate was acquired from Agar Scientific, UK. PMB sulfate powder was purchased from Santa Cruz Biotechnology Inc., Germany. FA sodium salt was obtained from Sigma‐Aldrich, UK. Milli‐Q water was obtained from a Merck Millipore Milli‐Q Integral water purification system with a minimum resistivity of 18.2 MΩ cm.

### PA Synthesis and Purification

PAs were synthesized in a Liberty Blue automated microwave peptide synthesizer (CEM, UK) using the standard Fmoc‐based solid phase peptide synthesis method. MBHA rink amide resin was used as the solid support and a 4:4:4 molar ratio of Fmoc‐amino acid/HOBt/DIC was used for the amino acid coupling cycles. Fmoc deprotections were performed with 20% v/v piperidine in DMF for 10 min twice. Palmitic acid was manually coupled to the peptide N‐terminus amine to obtain PAs. Coupling was performed overnight under the same conditions as the Fmoc‐amino acid. The final coupling was monitored with Kaiser test to confirm the lack of free amines. PAs were then cleaved from the resin with a mixture of TFA/TIS/H_2_O at a volume ratio of 95:2.5:2.5 for 2 h with simultaneous removal of all the protecting groups. The cleavage solution was then collected and the excess TFA was removed by rotary evaporation. Cold diethyl ether was added to precipitate the peptide product, which was then collected by centrifugation, washed again with cold diethyl ether, and dried under vacuum overnight.

PA purification was performed using an AutoPurification System (Waters, UK) equipped with a preparative reverse‐phase (RP) C18 column (XBridge, 130 Å, 5 µm, 30 × 150 mm, Waters, UK) and a gradient of H_2_O/ACN with 0.1% NH_4_OH as mobile phase. Fractions containing PAs were automatically collected when the exact mass signal was detected by a single quadrupole SQ Detector 2 mass detector (Waters, UK). The collected PA fractions were first concentrated by rotary evaporation, then lyophilized and stored at −20 °C until further use. Mass of PAs was confirmed by ESI‐MS (Agilent, UK) and purity of PAs was analyzed in an Alliance High‐Performance Liquid Chromatography system (Waters, UK), equipped with an analytical RP C18 column (XBridge, 130 Å, 3.5 µm, 4.6 × 150 mm, Waters, UK) and a UV detector set at the wavelength of 220 nm.

### CAC

CAC of PAs was determined using a previous reported method.^[^ ^22^ ^]^ Nile red, a hydrophobic solvatochromic dye, was dissolved in acetone and aliquoted to Eppendorf tubes before being left in a dark place at room temperature to generate dry films. PA solutions with various concentrations, ranging from 1 × 10^−7^ to 10 mg mL^−1^, were then prepared and added to Eppendorf tubes to dissolve the Nile red dry films to a final concentration of 1 × 10^−6^ m. After being aged overnight, each solution was analyzed on a LS55 fluorescence spectrometer (PerkinElmer, UK) with a fixed excitation wavelength of 550 nm. Fluorescence emission spectrum, ranging from 580 to 720 nm, was then acquired for each solution, with its maximum fluorescence intensity and the corresponding wavelength determined and plotted as function of the logarithm PA concentration. The CAC of PA was determined at the point where there was a sharp increase in the fluorescence intensity and a blueshift in the wavelength of the maximum emission.

### TEM

The morphology of PA assemblies was examined using TEM. 10 µL of PA solution at a concentration of 0.2 mg mL^−1^ was cast onto a carbon film‐coated copper grid with 400 square mesh (Agar Scientific, UK). Excess PA solution on the grid was removed with a piece of filter paper. 10 µL uranyl acetate solution, at a concentration of 2% v/v, was then loaded onto the grid to negatively stain the PA samples and the excess staining solution was removed after 30 s. Grids were allowed to dry at room temperature for at least 3 h before being imaged in a JEOL‐1230 TEM with an acceleration voltage of 80 kV. TEM images were recorded with a SIS Megaview III wide angle charge‐coupled device (CCD) camera.

### CD Spectroscopy

The secondary structure of PAs and PMB was characterized in a Chirascan CD spectrometer (Applied Photophysics, UK) in the far UV region (190–280 nm) at 25 °C. PA and PMB solutions were prepared at a concentration of 0.2 mg mL^−1^ in H_2_O and individually loaded into a 1 mm path length quartz cuvette. The ellipticity (mdeg) signal was recorded and then converted to mean residue ellipticity (deg cm^−2^ dmol^−1^) using the following equation

(3) $$\left[\theta \right]=\frac{\theta }{10\times c\times l\times n}$$

where *θ* is the recorded ellipticity in mdeg, *c* is the concentration of PA or PMB in M, *l* is the light path length of quartz cuvette in cm, and *n* is the number of amino acid residues in the PA or PMB molecules.

### Hydrogel Preparation

PMB Gels were initially prepared by first adding 100 µL of TPA solution in H_2_O, at a given concentration, onto the bottom of wells in 96‐well plates, followed by adding an equal volume of 2 mg mL^−1^ PMB solution, also in H_2_O, on top of the TPA solution. An opaque interface was immediately formed upon contact between the two solutions. The hydrogels were allowed to form overnight at 37 °C and were then rinsed with sterilized Milli‐Q water to ensure the removal of unreacted PMB and TPA. Similarly, Ca Gel and PMB+Ca Gel were also prepared by adding an equal volume of CaCl_2_ solution or 2 mg mL^−1^ PMB in CaCl_2_ solution, respectively. FA+PMB Gels were prepared by using TPA in a 2 mg mL^−1^ fusidic acid solution instead of pure H_2_O. Various concentrations of TPA and CaCl_2_ solutions were tested for optimizing the gel formulation. In addition to 96‐well plates, 1 mL transparent glass vials and 15 mL centrifuge tubes were also used to prepare the hydrogels in order to fulfil the requirements of the different characterization methods.

### SEM

The micro‐ and nanostructure of the hydrogels was examined by SEM. Hydrogels were first fixed with 2% v/v glutaraldehyde in PBS for 2 h at 4 °C, followed by washing with PBS for 3 times. Sequential dehydration of the hydrogels was then performed by immersion in increasing concentrations of ethanol (from 20% to 100%) for 20 min. Ethanol removal was performed using an EMS850 Critical Point Dryer (Electron Microscopy Science, USA) to avoid collapse of the gel structure. All hydrogels were coated with a gold layer (5–30 nm) using an Emitech SC7620 sputter coater (Quorum Technologies, UK) before being imaged under an Inspect F50 field emission gun SEM (FEI, Netherlands) with a beam voltage of 10 kV.

### Toluidine Blue Staining

Compositional differences between the two distinct layers of the hydrogels were assessed by cryostat and toluidine blue staining. Hydrogels were first fixed with 4% w/v paraformaldehyde in PBS for 2 h at 4 °C, followed by washing with PBS for 3 times. Sequential dehydration of gels was then performed with increasing concentrations of ethanol (from 20% to 100%). Sectioning of gels was performed using Cryostat (CM3050S, Leica, Germany). The sectioned gels were then stained with 1% w/v toluidine blue in H_2_O before being imaged under a microscope (DM2000, Leica, Germany) with 40× objective.

### EE% Study

The EE% of PMB in the hydrogels was quantified using the fluorescamine assay.^[^ ^29^ ^]^ Fluorescamine had the ability to react rapidly with primary amines of the PMB to form a fluorescence product. A series of PMB dilutions, with concentrations ranging from 0 to 2 mg mL^−1^, were prepared in water as standard solutions. The rinsing solutions of the hydrogels during the gel preparation were kept as sample solutions. 150 µL of the standard and sample solutions were first pipetted into 96‐well plates, followed by the addition of 50 µL of 3 mg mL^−1^ fluorescamine solution in acetone. The 96‐well plates were then placed on a microplate shaker for 1 min before being placed inside the FLUOstar OPTIMA microplate reader (BMG LABTECH, Germany). Fluorescence emission intensities were recorded at 460 nm with an excitation wavelength fixed at 400 nm. Data collected from the standard solutions were plotted as function of concentration to establish the standard curve (Figure S6, Supporting Information). The concentrations of sample solutions were then calculated based on the standard curve. The EE% of PMB in the hydrogels was quantified using the following equation

(4) $$EE\%=\frac{W-\left(C\times V\right)}{W}\times 100\%$$

where *C* is the concentration of sample solution calculated, *V* is the total volume of rinsing solution in the hydrogel preparation process, *W* is the total weight of PMB used to prepare the hydrogels.

### Rheology

The mechanical properties of the hydrogels were characterized by oscillatory rheology using a DHR‐3 Rheometer (TA Instruments, USA) equipped with an 8 mm diameter parallel plate geometry. Amplitude sweep was first performed in the 0.01–10% strain range and with a constant frequency of 1 Hz. *G′* (storage modulus) and *Gʺ* (loss modulus) of the hydrogels were monitored. A fixed strain, up to which *G′* and *Gʺ* showed no variation (in the linear viscoelastic region) during the amplitude sweep, was then used as input for further dynamic frequency sweep performed in the 0.1–100 Hz range. Temperature was maintained at 37 °C during all measurements.

### PMB Release Study

The release kinetics of PMB from the hydrogels was investigated at 37 °C by first preparing the hydrogels at the bottom of 15 mL centrifuge tubes and then adding 1 mL of the release media on the top of hydrogels (Figure S8, Supporting Information). 1× PBS (pH 7.4) buffer was chosen as the release media and 37 °C was used as the experimental temperature to more closely mimic the physiological environment. 150 µL of release media were sampled at 0, 0.5, 1, 2, 4, 8, 12, 24, 48, 72, and 120 h with replenishment of an equal volume of fresh PBS. Concentration of PMB in the sampled release media was determined using the fluorescamine assay. Cumulative PMB release was then calculated as percentage and plotted as function of release time. Release curves were obtained by fitting the data by appropriate models in Origin and release kinetics were determined from the fitting equations.

### Bacteria Strains and Media

Bacterial type strains, *E. coli* (NCTC 25922), *A. baumannii* (NCTC 19606), and *S. aureus* (NCTC 25923) were obtained from the National Collection of Type Cultures (Public Health England, UK). *P. aeruginosa* PA14 isolate was sourced from Barts Health NHS Trust. Bacterial culture media were purchased from Oxoid Ltd., UK, prepared and autoclaved according to the manufacturer's instructions. Isolates were cultured in Mueller Hinton II (MH‐II) Broth under constant shaking at 1100 rpm at 37 °C to mid‐exponential growth phase (18 h).

### Sustained Bacterial Inhibition Assay

The sustained bacterial inhibition assay of antibiotic (PMB and FA)‐loaded hydrogels was performed using a previously reported disk transfer and diffusion method.^[^ ^34^ ^]^ Bacterial suspensions of each isolate were prepared in MH‐II Broth and adjusted to 0.5 McFarland standard. An even lawn of bacterial suspension was then swabbed onto MH‐II agar plates (Oxoid, UK). The sensitivity of each bacterial isolate to inhibition with different antibiotics was first examined using disks (Oxoid, UK) that were loaded with 10 µL of a series of antibiotic solutions (10 times dilutions with concentrations ranging from 20 000 to 2 µg mL^−1^). Sustained antibiotic release from the hydrogels was then examined via a transfer experiment in which disks were loaded with either hydrogel containing antibiotic or 10 µL antibiotic solution containing an amount equivalent to that loaded in the hydrogels. After incubation for 1.5 h on MH‐II agar plates, disks were transferred to a second plate with an addition of 10 µL sterile PBS to each disk for simulating the continuous release of antibiotics. The transfer process was repeated every 1.5 h. All the disks were disposed after 24 h and the plates were then incubated at 37 °C in room air for 18 h. Images of the plates were taken and the inhibitory zones against bacterial growth were measured in millimeter using electronic digital caliper.

### *G. Mellonella* Burn Wound Infection Model

The in vivo antimicrobial efficacy of antibiotic loaded hydrogels was performed using a previously established *G. mellonella* burn wound infection model.^[^ ^40^ ^]^ TruLarv *Galleria* were purchased from Biosystems Technology (UK) and stored at 4 °C before use. Their bodies were first sterilized with 70% ethanol and then sorted into Petri dishes lined with Whattman filter paper (Fisher Scientific, UK). A heated metal spatula was applied onto the back of *Galleria* for 4 s to achieve a consistent burn area of ≈2 mm^2^. At least 20 min after the burning process, 10 µL of 10 times dilution of overnight cultured bacterial inoculum was dropped onto the burn wound for infection. Hydrogels were then applied to completely cover the burn area on the back of *Galleria* (Figure S13, Supporting Information). The *Galleria* were placed in individual wells of a 6‐well plate to avoid detaching the hydrogel due to their movement interactions, followed by incubation at 37 °C for 120 h. The color of *Galleria* was examined by observation and the state of *Galleria* (dead or alive) was determined by pricking them with a plastic pipette tip. Mortality of *Galleria* was recorded by a color change, from golden to black or dark brown, and lack of movement when pricked. Triplicate set of ten *Galleria* were tested in each group. Pooled Kaplan–Meier survival curves were plotted to visualize data. Statistical significance of differences was determined using a Logrank (Mantel–Cox) test with *p* < 0.01 considered significant.

### Statistical Analysis

Numerical data were expressed as mean ± standard deviation (SD). All experiments were repeated at least 3 times. Statistical analysis was performed using SPSS software (IBM, USA). Statistical significance of differences was determined using independent‐sample *t*‐test (*p < 0.05, **p < 0.01, and ***p < 0.001), unless otherwise specified.

## Conflict of Interest

The authors declare no conflict of interest.

## Supporting information

Supporting Information

## Data Availability

The data that support the findings of this study are available in the Supporting Information of this article.
