# Supplementary material for: Polymyxin B‐Triggered Assembly of Peptide Hydrogels for Localized and Sustained Release of Combined Antimicrobial Therapy
Source: Adv Healthc Mater. 2021 Sep 14;10(22):2101465. doi: 10.1002/adhm.202101465 (PMC11469027; doi:10.1002/adhm.202101465)
Supplement: Supplementary file 1 — Supporting Information [file ADHM-10-2101465-s001.pdf]

WILEY-VCH

© 2021 Wiley-VCH GmbH

ADVANCED HEALTHCARE MATERIALS

Supporting Information

for *Adv. Healthcare Mater.,* DOI: 10.1002/adhm.202101465

Polymyxin B-Triggered Assembly of Peptide Hydrogels for Localized and Sustained Release of Combined Antimicrobial Therapy

Yejiao Shi, David W. Wareham, Yichen Yuan, Xinru Deng, Alvaro Mata, and Helena S. Azevedo*

Supporting Information

Polymyxin B-Triggered Assembly of Peptide Hydrogels for Localized and Sustained Release of Combined Antimicrobial Therapy

*Yejiao Shi,[1] David W. Wareham,[2,3] Yichen Yuan,[1] Xinru Deng,[1] Alvaro Mata,[4,5,6] and Helena S. Azevedo[1]**

[1]School of Engineering and Materials Science & Institute of Bioengineering, Queen Mary, University of London, London E1 4NS, UK

[2]Center for Immunobiology, The Blizard Institute, Barts and The London, School of Medicine and Dentistry, Queen Mary, University of London, London E1 2AT, UK

[3]Barts Health NHS Trust, London E1 2AT, UK

[4]School of Pharmacy, University of Nottingham, Nottingham NG7 2RD, UK

[5]Department of Chemical and Environmental Engineering, University of Nottingham, Nottingham NG7 2AT, UK

[6]Biodiscovery Institute, University of Nottingham, Nottingham NG7 2RD, UK

*E-mail: h.azevedo@qmul.ac.uk

Contents

**Figure S1**	Characterization of TPA ($C_{15}H_{31}$CONH-ETE-$CONH_2$)
**Figure S2**	Characterization of EPA ($C_{15}H_{31}$CONH-EEE-$CONH_2$)
**Figure S3**	Characterization of BPA ($C_{15}H_{31}$CONH-VVVAAAEEE-$CONH_2$)
**Figure S4**	Characterization of the commercially purchased PMB
**Figure S5**	CAC determination for TPA and BPA
**Figure S6**	Standard curve of PMB
**Figure S7**	Mechanical stiffness of the three representative PA hydrogels
**Figure S8**	Images of the hydrogels over the release period (120 h, 5 days).
**Figure S9**	Bacterial inhibition activity of PMB solutions and hydrogels against *P. aeruginosa* PA14
**Figure S10**	Bacterial inhibition activity of PMB solutions and hydrogels against *E. Coli*
**Figure S11**	Bacterial inhibition activity of PMB solutions and hydrogels against *A. Baumannii*
	Safety profile of TPA and PMB solutions on *Galleria Mellonella*
**Figure S12**	
**Figure S13**	Representative images of the *Galleria Mellonella* immediately after the various procedures
**Table S1**	Ability of various polyanionic solutions with high viscosity to form hydrogels when mixed with PMB

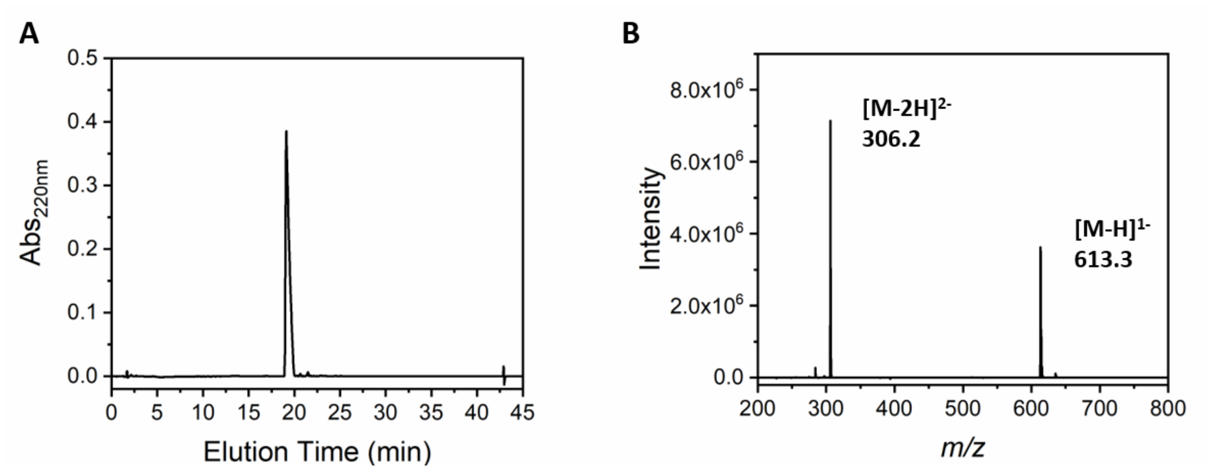

Figure S1. Characterization of TPA ($C_{15}H_{31}$CONH-ETE-CONH$_2$): (A) analytical RP-HPLC trace under the gradient of 98% to 0% H_2O (2% to 100% ACN) with 0.1% NH_4OH from 5 to 35 min showing high purity; **(B)** ESI-MS spectrum showing the expected molecular mass ($C_{30}H_{54}N_4O_9$, Mw: 614.77 g/mol).

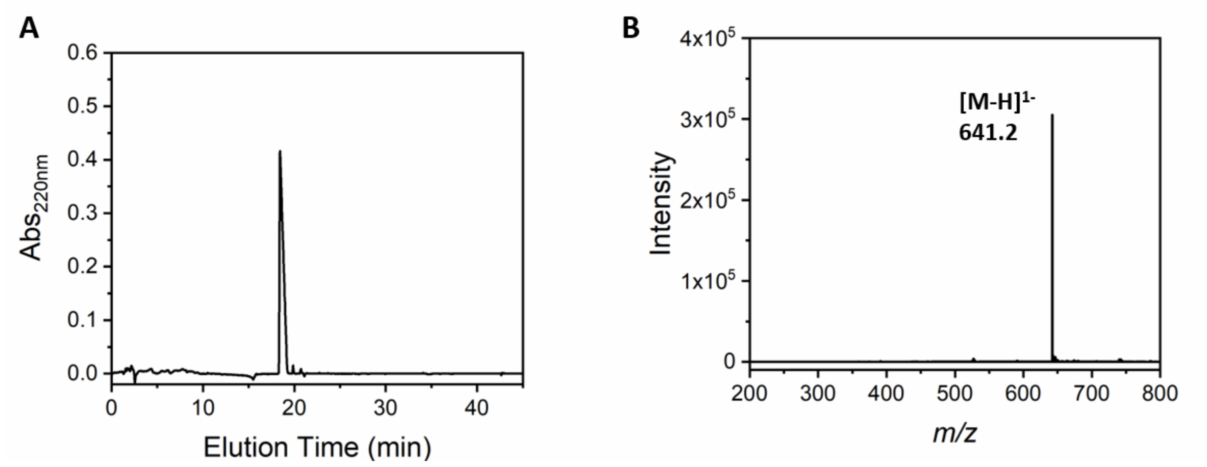

Figure S2. Characterization of EPA ($C_{15}H_{31}$CONH-EEE-CONH$_2$): (A) analytical RP-HPLC trace under the gradient of 98% to 0% H_2O (2% to 100% ACN) with 0.1% NH_4OH from 5 to 35 min showing high purity**; (B)** ESI-MS spectrum showing the expected molecular mass ($C_{31}H_{54}N_4O_{10}$, Mw: 642.78 g/mol).

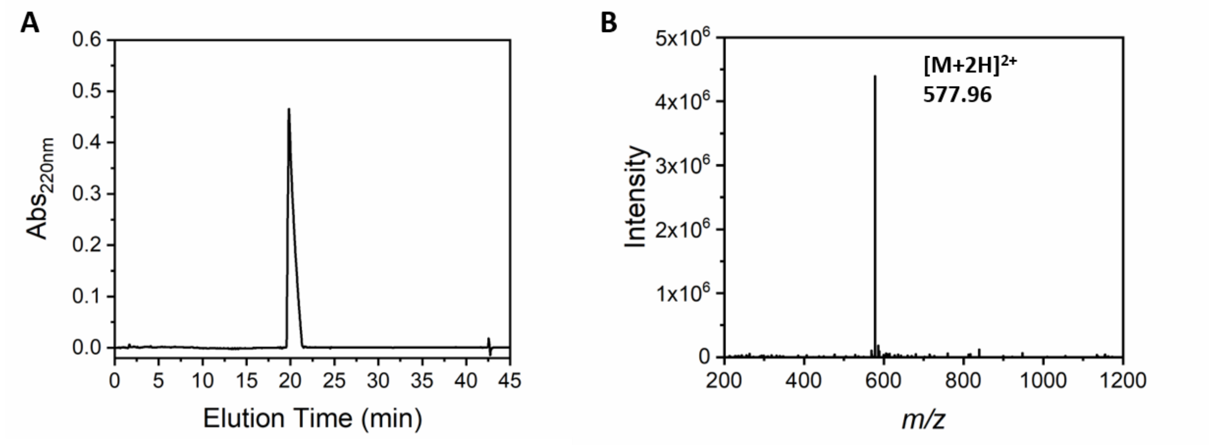

Figure S3. Characterization of BPA ($C_{15}H_{31}$CONH-VVVAAAEEE-$CONH_2$): (A) analytical RP-HPLC trace under the gradient of 98% to 0% H_2O (2% to 100% ACN) with 0.1% NH_4OH from 5 to 35 min showing high purity; **(B)** ESI-MS spectrum showing the expected molecular mass ($C_{55}H_{96}N_{10}O_{16}$, Mw: 1153.41 g/mol).

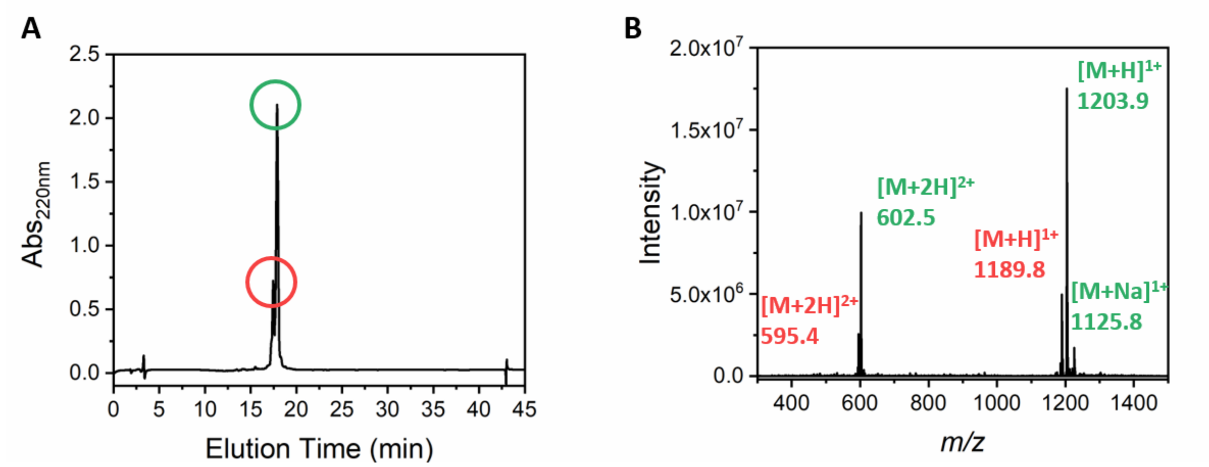

Figure S4. Characterization of the commercially purchased PMB: (A) analytical RP-HPLC trace of PMB under the gradient of 98% to 0% H_2O (0.1% TFA) from 5 to 35 min, showing two peaks and indicating that the purchased PMB contains a mixture of PMB1 (labeled with green circle) and PMB2 (labeled with red circle); **(B)** ESI-MS spectrum of PMB showing the expected molecular mass and confirming the presence of both PMB1 ($C_{56}H_{98}N_{16}O_{13}$, Mw: 1203.50 g/mol) and PMB2 ($C_{55}H_{96}N_{16}O_{13}$, Mw: 1189.47 g/mol) in the commercial PMB product.

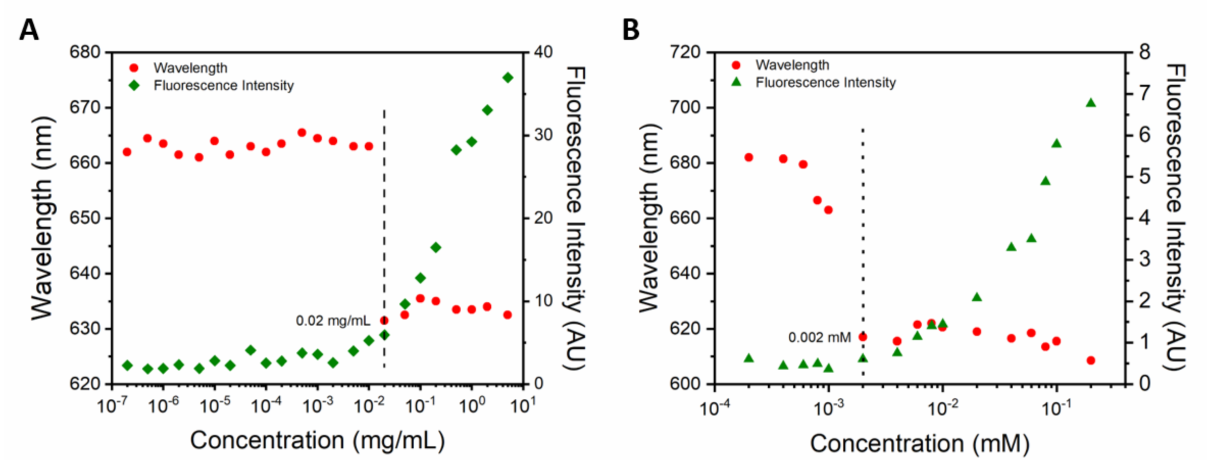

Figure S5. CAC determination for TPA and BPA: maximum fluorescence emission wavelength and intensity of Nile red as function of PA concentration to determine the CAC. **(A)** CAC of TPA at 0.02 mg/mL**; (B)** CAC of BPA at 0.002 mM (0.002 mg/mL).

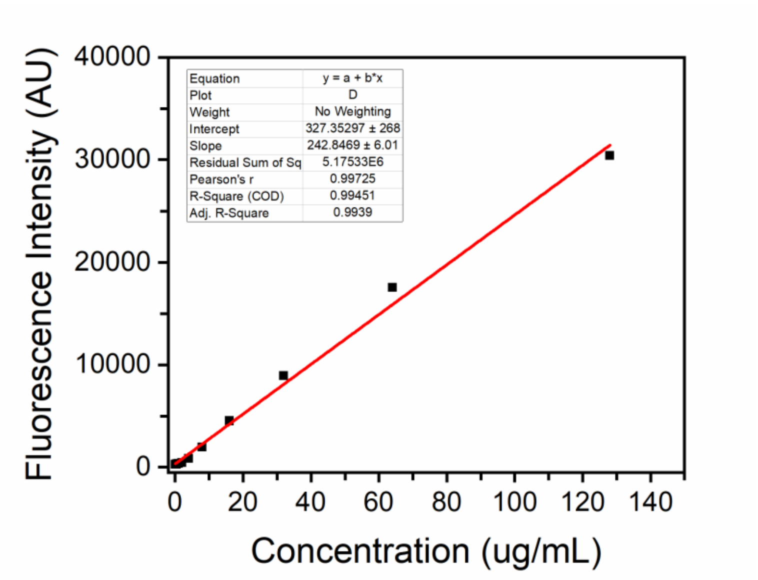

Figure S6. Standard curve of PMB: fluorescence intensity of fluorescamine measured as function of the concentration of PMB.

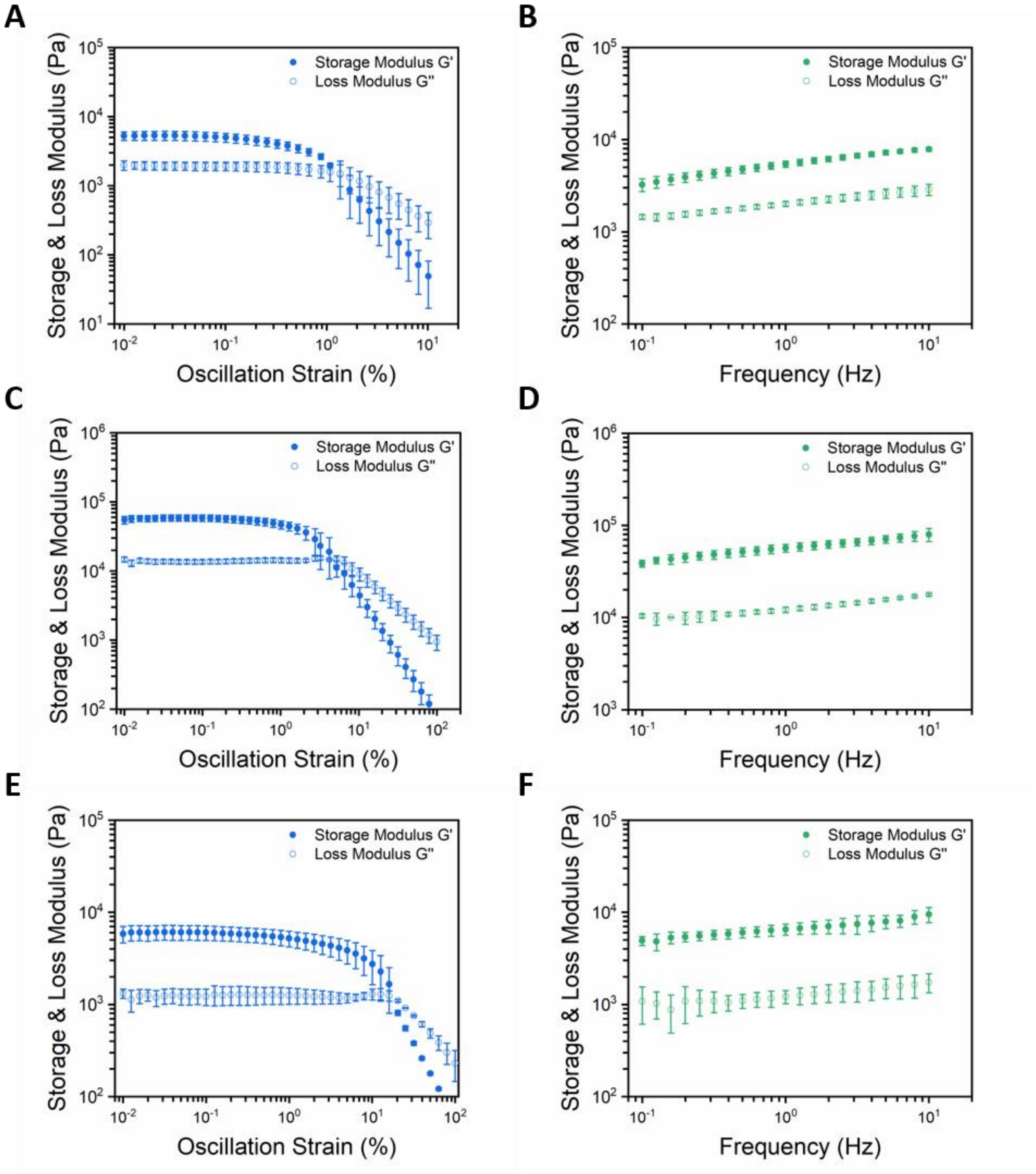

Figure S7. Mechanical stiffness of the three representative PA hydrogels: amplitude sweep of the PMB Gel (**A**), PMB+Ca Gel (**C**), and Ca Gel (**E**); frequency sweep of the PMB Gel (**B**), PMB+Ca Gel (**D**), and Ca Gel (**F**).

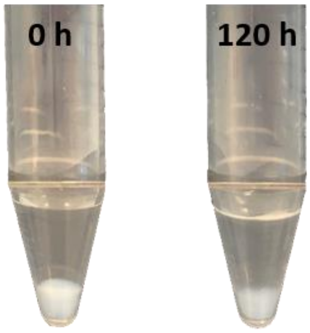

Figure S8. Images of the hydrogels over the release period (120 h, 5 days): The PMB-triggered PA hydrogels were prepared at the bottom of the 15 mL centrifuge tubes with 1 mL PBS added on top for performing the release experiments.

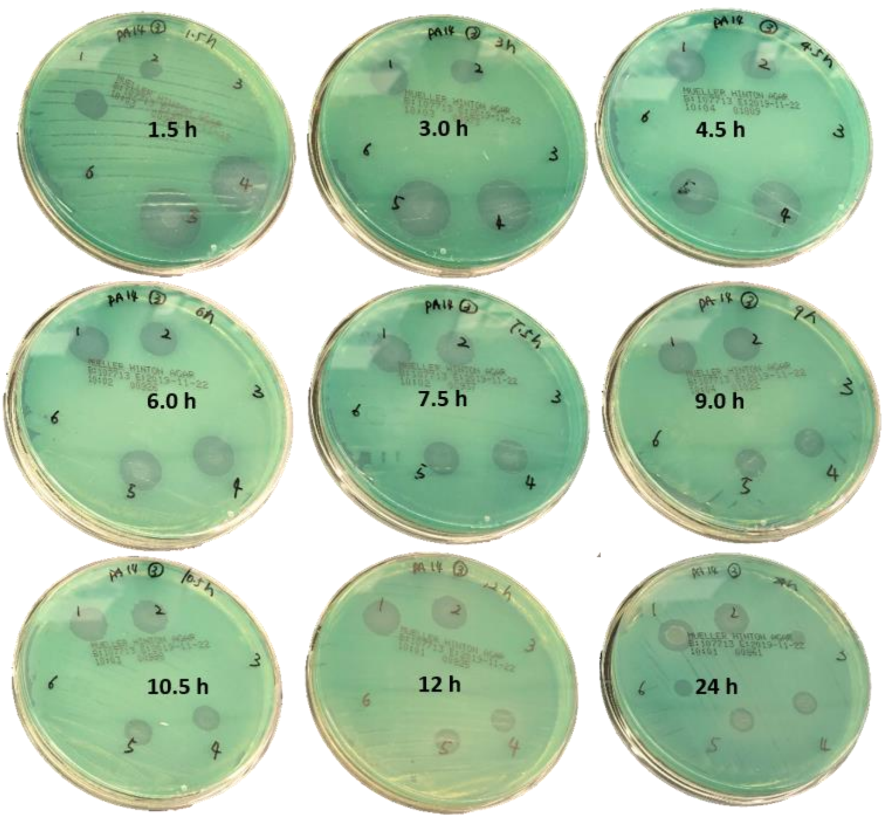

Figure S9. Bacterial inhibition activity of PMB solutions and hydrogels against *P. aeruginosa* PA14: pictured agar plates showing antimicrobial activities of 1-PMB Gel, 2-PMB+Ca Gel, 3-Ca Gel, 4-PMB Solution, 5-PMB+Ca Solution, 6-

PBS with their loaded discs being diffused and transferred onto *P. aeruginosa* PA14 suspension covered agar plate every 1.5 h over a 24 hour period (visible zone shown for 6-PBS at 24 h is due to the removal of the disc at the end of the experiment).

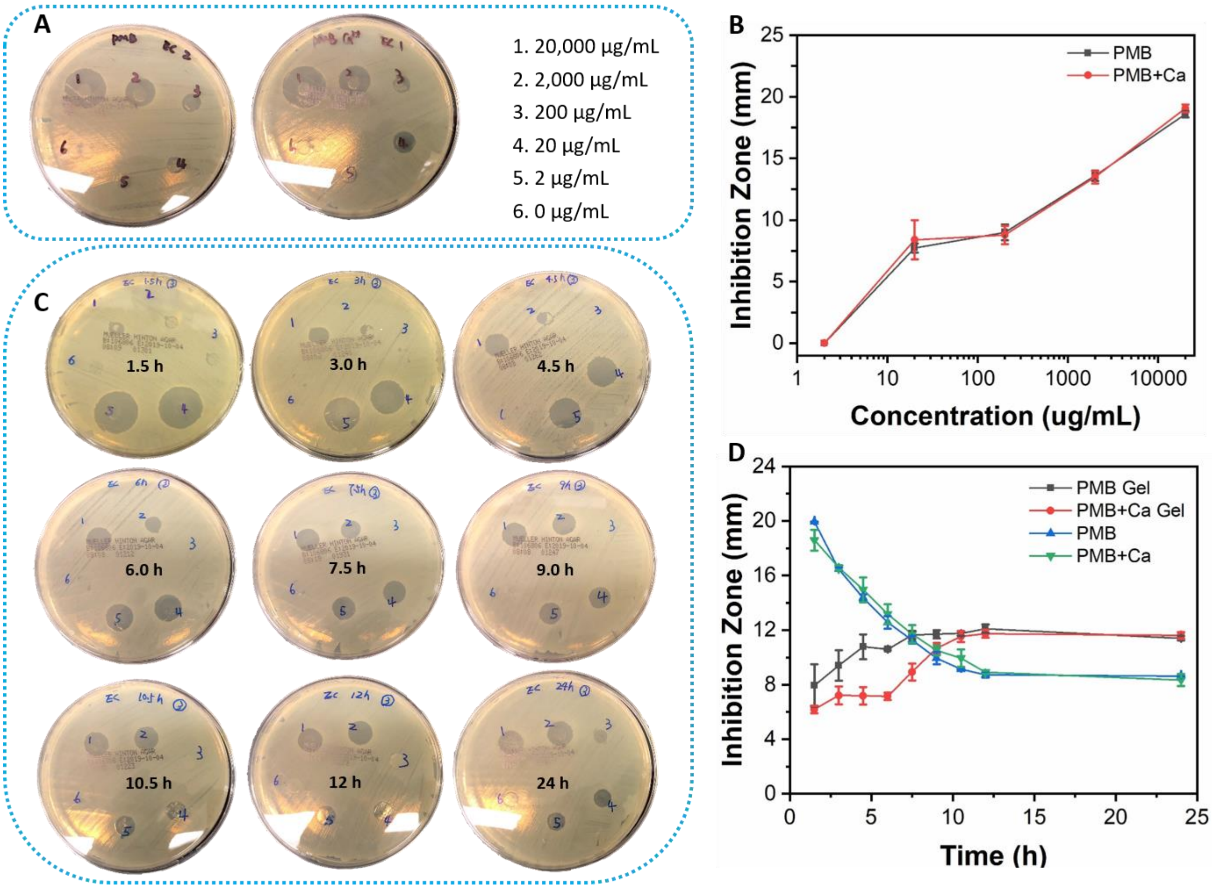

Figure S10. Bacterial inhibition activity of PMB solutions and hydrogels against *E. coli*: (**A**) pictured agar plates showing susceptibility of *E.coli* to PMB and PMB+Ca solutions examined by dose response experiments; (**B**) Inhibition zone changes in response to the different concentrations of PMB; (**C**) pictured agar plates showing antimicrobial activities of 1-PMB Gel, 2-PMB+Ca Gel, 3-Ca Gel, 4-PMB Solution, 5-PMB+Ca Solution, 6-PBS with their loaded discs being diffused and transferred onto *E. coli* suspension swabbed agar plate every 1.5 h over a 24 hour period (visible zone shown for 6-PBS at 24 h is due to the removal of the disc at the end of the experiment); (**D**) Inhibiton zone changes over time for the different formulations of PMB. Ca Gel and PBS displayed no bacterial inhibition activity against *E. coli* and no inhibition zones were observed. Data are given as mean ± SD (n = 3).

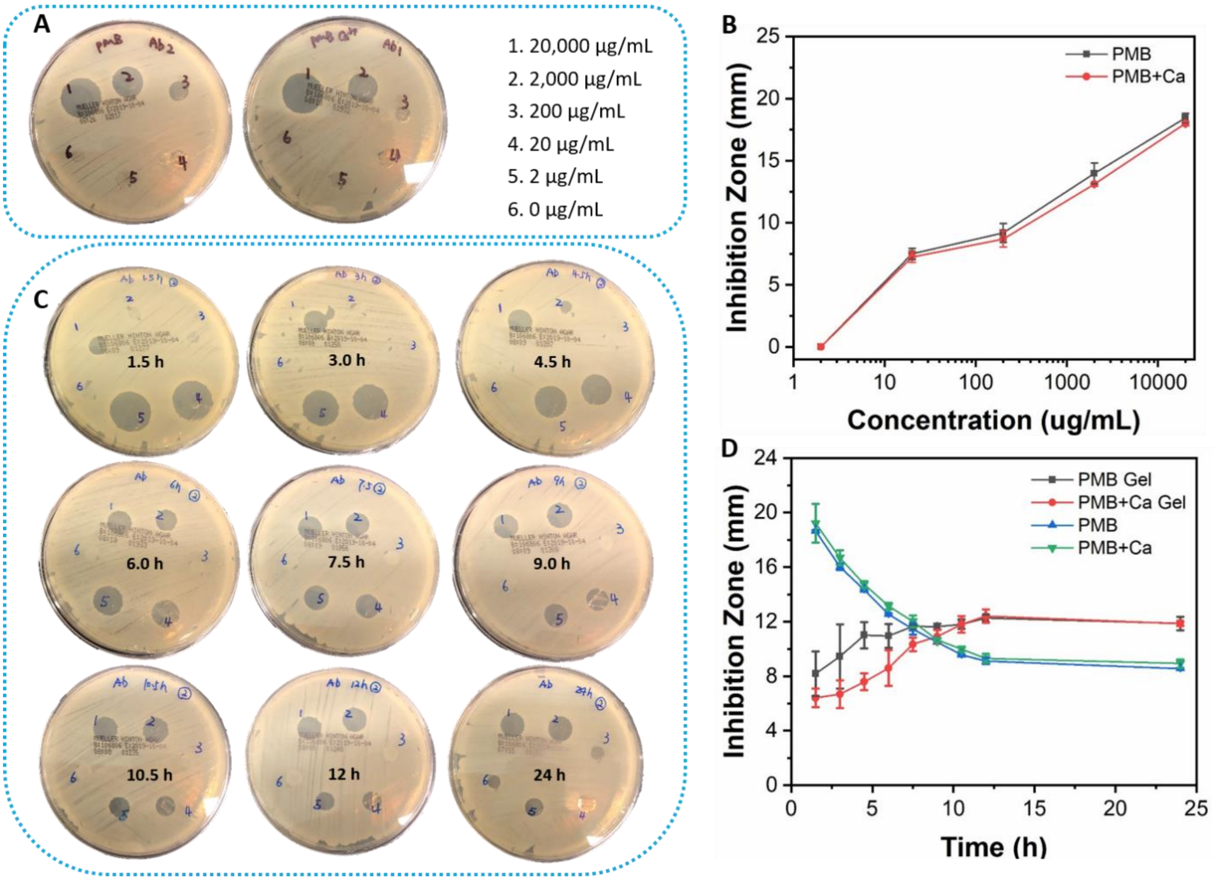

Figure S11. Bacterial inhibition activity of PMB solutions and hydrogels against *A. baumannii*: (**A**) pictured agar plates showing susceptibility of *A. baumannii* to PMB and PMB+Ca solutions examined by dose response experiments; (**B**) Inhibition zone changes in response to the different concentrations of PMB; (**C**) pictured agar plates showing antimicrobial activities of 1-PMB Gel, 2-PMB+Ca Gel, 3-Ca Gel, 4-PMB Solution, 5-PMB+Ca Solution, 6-PBS with their loaded discs being diffused and transferred onto *A. baumannii* suspension covered agar plate every 1.5 h over a 24 hour period (visible zone shown for 6-PBS at 24 h is due to the removal of the disc at the end of the experiment); (**D**) Inhibiton zone changes over time for the different formulations of PMB. Ca Gel and PBS displayed no bacterial inhibition activity against *E. coli* and no inhibition zones were observed. Data are given as mean ± SD (n = 3).

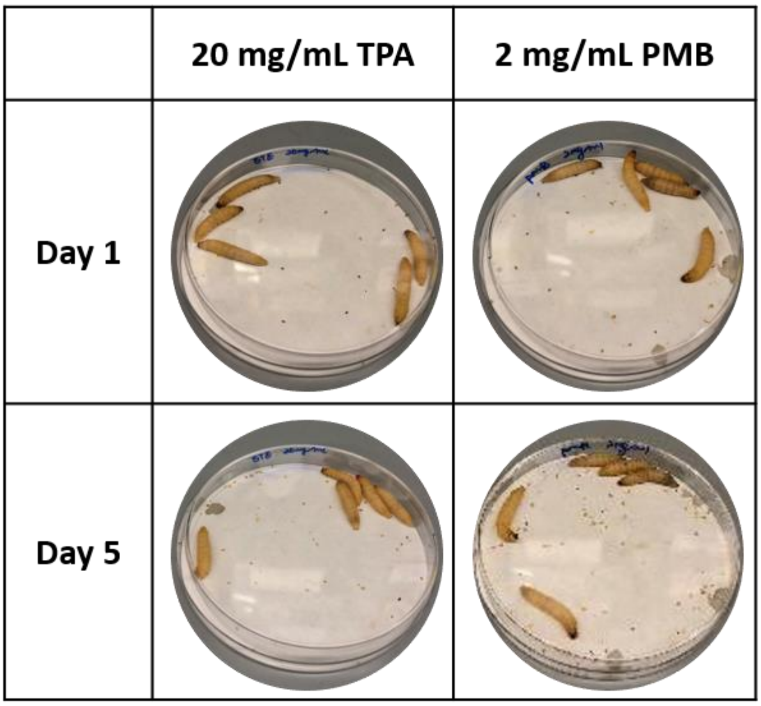

Figure S12. Safety profile of TPA and PMB solutions on *Galleria Mellonella*: Images taken at different periods of time after injection of 10 µL TPA (20 mg/mL) and PMB (2 mg/mL) solutions into the *Galleria Mellonella*, using the BD Micro-Fine Insulin Syringe (0.3 mL) with Needle (30 G), with no obvious toxicity observed at day 1 and day 5.

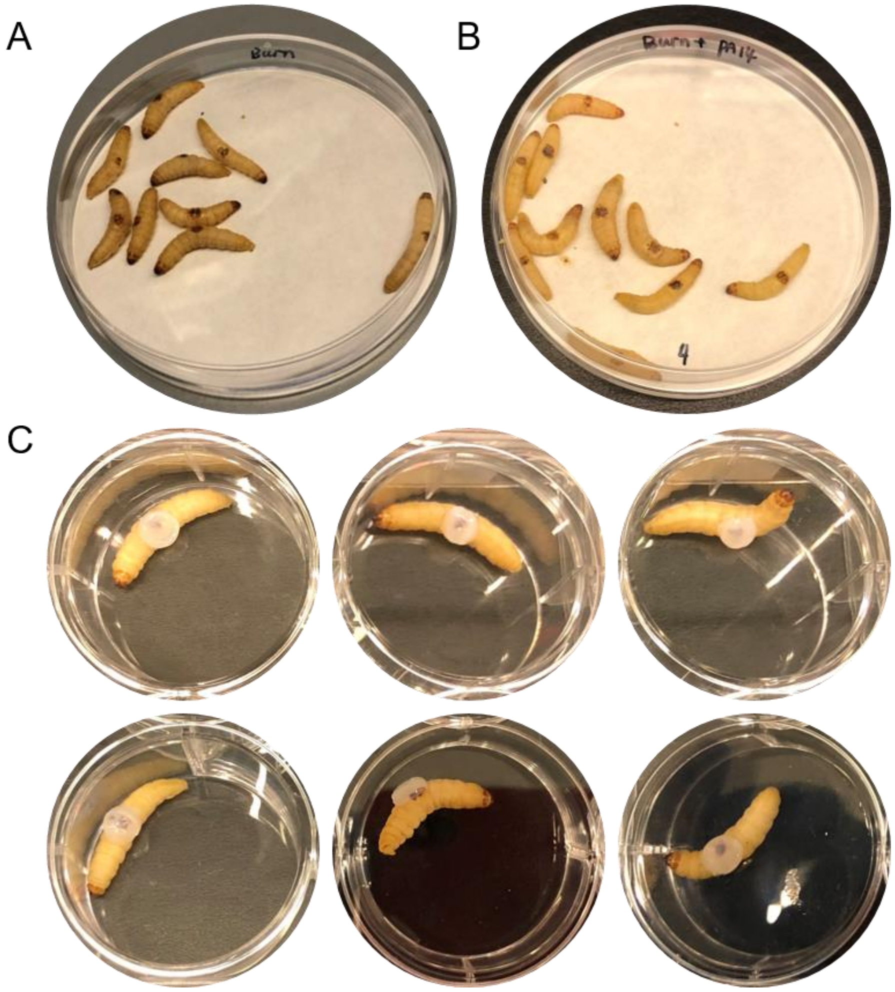

Figure S13. Representative images of the *Galleria Mellonella* immediately after the various procedures: (**A**) after burn; **(B)** after burn and bacteria inoculation; **(C)** with hydrogel onto the infected burn wound.

Table S1. Ability of various polyanionic solutions with high viscosity to form hydrogels when mixed with PMB: the names and chemical structures of different polyanions tested; pictures showing the initial viscosity of different polyanionic solutions (20 mg/mL), when they are mixed with equal volume of 2 mg/mL PMB solution, and after being incubated at 37 ℃ overnight.

**Polyanions**	**Chemical Structure**	**Polyanionc Solution (20 mg/mL)**	**+ PMB Solution (2 mg/mL)**	**Overnight Incubation (37 ℃)**	**Observations**
**Alginate**	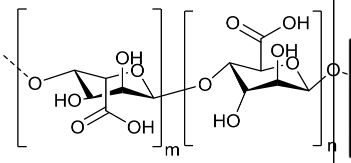	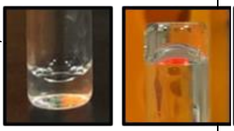	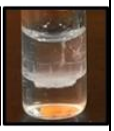	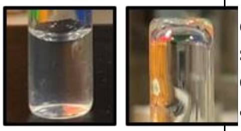	Opaque interface appeared with addition of PMB solution, but disappeared after overnight incubation; viscosity decreased.
**Hyaluronic Acid**	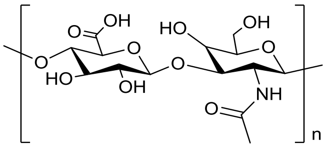	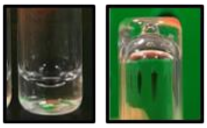	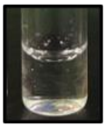	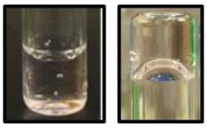	No significant changes were observed with addition of PMB solution.
**Polyacrylic Acid**	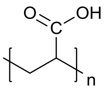	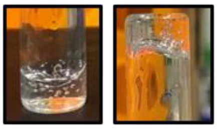	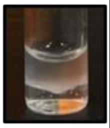	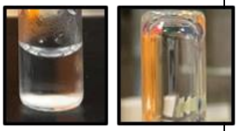	Opaque interface appeared with addition of PMB solution, but disappeared after overnight incubation; viscosity decreased.
